# Case Study on the Genetic Parameters and Possibilities of Selecting Gilts for Traits Monitored in the Performance Test

**DOI:** 10.3390/vetsci12050500

**Published:** 2025-05-20

**Authors:** Nenad Stojiljković, Čedomir Radović, Marija Gogić, Vladimir Živković, Aleksandra Petrović, Krstina Zeljić Stojiljković, Dubravko Škorput

**Affiliations:** 1 Institute for Animal Husbandry, Autoput 16, 11080 Belgrade, Serbia; 2 Faculty of Agriculture, University of Zagreb, Svetošimunska Cesta 25, 10000 Zagreb, Croatia

**Keywords:** pigs, field test, modeling, heritability, backfat thickness, MLD

## Abstract

This research examined the phenotypic and genotypic variability of traits assessed in the gilt performance test and their subsequent impact on gilt selection. The traits evaluated in the gilt performance test were analyzed on two pig farms over a period of 3 consecutive years. A total of 3664 gilts were included in the research. At the end of the test, body weight, backfat thickness (BF1 and BF2), and longissimus dorsi muscle depth (MLD) were measured using an ultrasound device. The following breeds were evaluated on the farms: Landrace (L)–1981 gilts, Large White (LW)–1344 gilts, and Duroc (D)–339 gilts. In the analyzed population, direct genetic effects accounted for 0.2647 of the total variation in age at the end of the test (AET). Heritability coefficients of 0.37 for BF1 and 0.35 for BF2 indicate that these traits are highly heritable in the studied population. On the other hand, the heritability coefficient for the depth of MLD, which is 0.23, places this trait in the group of medium heritable traits. High heritability coefficients of these traits indicate great potential for genetic improvement through selection. The use of well-designed selection programs aimed at these traits can significantly accelerate the genetic improvement of the population and have an impact on the economic profit of pork production.

## Simple Summary

In the Republic of Serbia, selecting gilts for breeding is mostly based on their phenotypic characteristics. Because the estimation of breeding value is not done systematically, genetic parameters remain unknown although it is well known that a genetic improvement of high heritability traits in gilts can be achieved relatively easily by selection of these traits. Thus, it is important to precisely estimate heritability coefficients of economically important traits monitored in a performance test of gilts. This is essential since the effect of selection depends on the precision of estimation of heritability coefficients. Effects of selection are directly proportional to the accuracy of estimation of breeding value i.e., the estimation of heritability coefficients which precede. These findings highlight the importance of the estimation of variance components for production traits of gilts and consequently their effect on genetic improvement of economically important traits in the pig meat industry.

## 1. Introduction

The management of gilts on farms is crucial for successful herd management as it directly affects future pig meat production results [1]. The performance test is a key selection tool in pig breeding [2]. In Serbia, performance testing of gilts is mandatory for all breeders engaged in the production and sale of breeding stock. The test is conducted on the farm of origin and includes gilts selected based on current breeding criteria, such as correct teat placement and pedigree from the Main Herdbook. At the end of the test, body weight, backfat thickness, loin muscle depth, and exterior traits are evaluated. The primary aim of selection on pig farms is to enhance animal performance by increasing the prevalence of favorable genes. Utilizing performance test results for selection enables the genetic improvement of key economic traits in pork production, such as daily gain, backfat thickness, and loin muscle depth, achieving annual progress of 2–3% [3].

For the successful implementation of selection with aim to increase pork production, it is crucial to understand the influence of external factors that affect the quantity and quality of meat, such as housing, diet, season and other environmental conditions. These external factors influence the expression of genetic potential; for example, inadequate nutrition can reduce the growth rate and muscle development, while poor housing conditions or extreme temperatures may increase stress in animals, negatively affecting fat deposition and overall carcass quality. The initial step in estimating heritability coefficients involves identifying and quantifying the impact of external environmental factors. The effect of the testing year on backfat thickness and muscle depth in F1 crossbred gilts (PLW × PL and PL × PLW) from Large White and Landrace breeds was investigated by [4]; their study demonstrated a marked and substantial impact of the year in which testing was conducted on the P2 backfat thickness, measured 3 cm laterally from the midline behind the last rib. Similarly, in their research the authors [5] reported a marked difference in the final body weight of gilts among herds across all analyzed years. Furthermore, their research confirmed that the testing period exerted a notable effect on all assessed traits. The carcass quality of gilts in the field test was analyzed in the research [6], with the study carried out at two production locations, where the animals reached approximately 209 days of age, a body weight close to 104 kg, and an average daily growth rate of around 0.49 kg. An ultrasound device (Piglog 105) was used to measure backfat thickness, revealing an average of 20.09 mm in the loin region (BF1) and 15.25 mm in the back region (BF2), while the longissimus dorsi depth (MLD) averaged 45.25 mm. The impact of external factors on trait variability observed during the performance test has been examined in several studies, including those by [7,8,9,10].

Economically significant traits observed in the performance test of gilts’ daily life gain (DLG), BF, and MLD are traits with high and medium heritability and can be relatively easily improved through individual selection. Breeding and selection programs include performing a performance test for the listed traits by measuring weight, while backfat thickness and muscle depth are measured with an ultrasound machine. Several studies have explored the heritability coefficients of traits assessed in the gilt performance test, including research by [2,7,8,10,11,12,13,14].

In the Republic of Serbia, the methodology for gilt performance testing has been developed and implemented in practice in the field. However, the genetic parameters of the monitored traits are largely unknown. This paper analyzes the phenotypic variability and estimates the genetic parameters of traits recorded during the performance testing of gilts on farms in the Republic of Serbia, with the aim of determining their heritability coefficients. The selected traits, such as growth rate, backfat thickness, and age at the end of test, are key indicators of production efficiency and carcass quality in pigs. Understanding the genetic basis of these traits is essential for improving breeding programs and enhancing pork production outcomes. In addition, the estimation of genetic parameters can significantly affect the selection response for these traits and help define further selection strategies and methods to be applied.

## 2. Materials and Methods

Over the course of three consecutive years, traits monitored during the performance test of gilts were analyzed at two pig farms, with a total of 3664 gilts included in the study. Only sires with a minimum of 10 daughters were included in the analysis. At the end of the test, measurements for body weight, backfat thickness (BF), and muscle depth (MLD) were taken using an ultrasound device. The study analyzed data from a total of 3664 gilts belonging to three genetic groups: Landrace (L), Large White (LW), and Duroc (D). The first farm tested 1380 animals (805 L, 341 LW, 87 D), while the second farm tested 2284 animals (1176 L, 1003 LW, 252 D). Based on the number of animals and breed composition, the groups were formed as follows: Group 1–Landrace, Group 2–Large White, and Group 3–Duroc. The testing was conducted over three consecutive years: 896 gilts were tested in the first year, 1165 in the second year, and 1603 in the third year.

This study analyzed the following traits: age at the end of the test (AET), backfat thickness between the third and fourth lumbar vertebrae, measured 7 cm laterally from the dorsal midline (BF1), fat thickness between the third and fourth ribs at the back, also 7 cm lateral to the midline (BF2), and the depth of the dorsal muscle (MLD). Phenotypic measurements and variation of the analyzed characteristics were evaluated through a linear model approach, utilizing the General Linear Model (GLM) procedure implemented in the SAS statistical software, version 9.4. [15]. The following models were used for the analysis:

$$Y_{ijk}=\mu +F_{i}+G_{j}+R_{k}+b_{1}\;(X_{1}-{\bar{x}}_{1})+\varepsilon_{ijk}$$

To evaluate the influence of fixed factors on backfat thickness (BF1 and BF2) and muscle depth in gilts undergoing performance testing, statistical analysis was conducted based on the first applied model, with differences considered meaningful at the *p* < 0.05 level. In this model, *Y_ijk_* represents the observed trait value, *μ* is the overall population mean, *F* denotes the farm effect, *G* represents the testing year, and *R* corresponds to the animal’s genotype. The parameter *b*_1_ is the linear regression coefficient for final body weight, while ε accounts for the random error. The indices *i*, *j*, and *k* are used to denote the farm (*i* = 1, 2), the year of testing (*j* = 1, 2, 3), and the genotype of the head (*k* = 1, 2, 3), respectively.

*Y_ijk_* = *µ* + *F_i_* + *G_j_* + *R_k_* + *ε_ijk_*

A second analytical model, comparable to the initial approach, was applied to evaluate the influence of fixed factors (*p* < 0.05) on the test completion age of gilts subjected to performance evaluation. In this model, *Yijk* represents the observed value of the trait for the *i* farm, the *j* year of testing and the *k* genotype. The symbol *µ* indicates the general population average, *F* indicates the farm, *G* the year of testing, and *R* the genotype of the head, while *ε_ijk_* represents the random error. The indices *i*, *j*, and *k* are used to denote the farm (*i* = 1, 2), the year of testing (*j* = 1, 2, 3), and the genotype of the animal (*k* = 1, 2, 3), respectively.

The model’s defined factors related to test age excluded the linear dependence of body mass on age variation, since this parameter had already been standardized to an age corresponding to a 100 kg body weight. The standardization was carried out according to the equation below:

*Corrected final age* = (*True age at measurement* × 100 kg)/*Final body weight*

The variances and co-variances of the examined traits were estimated using the VARCOMP procedure and the maximum likelihood (ML) method within the SAS software package [15]. This procedure required adjusting the analyzed traits (AET, BF1, BF2, and MLD) to a standardized body weight of 100 kg, which represents the target weight at the end of the test. The formula for correcting age at 100 kg is provided in this section as part of the explanation of the systematic model used to assess the phenotypic variability of age at the end of the test. Traits such as backfat thickness (BF1 and BF2) and longissimus muscle depth (MLD) at the end of the test were adjusted using the base index method, following the equations outlined below:

*Yi* = *a* + *bXi*

where:

*Yi*—expected value (predictor variable) of an individual’s trait

*a*—intercept *b*—linear regression coefficient,

*Xi*—the observed value of the predictor variable (weight) for the individual whose dependent variable is being adjusted

*C* = (*a* + *b* × 100)/*Yi*

where:

*C*—base index (coefficient for correction),

100—the reference value of the independent variable used for the adjustment of the dependent variable.

Corrected backfat thickness values for each gilt were obtained by multiplying the established base indices with the actual measured backfat thickness values. These corrected values were used to analyze the production results of each animal. The variances of the observed traits were calculated using the following model:

*Y_ijkl_* = *µ* + *F_i_* + *G_j_* + *R_k_* + *o_l_* + *ε_ijkl_*

where:

*Y_ijkl_*—phenotypic manifestation of the examined trait, *µ*—general population average, *F_i_* = fixed effect of farm, *G_j_* = fixed effect of year of testing, *R_k_* = fixed effect of individual’s genotype, *o_l_* = random effect of boar sire, *e_ijkl_* = random error.

Heritability was estimated through the interclass correlation of half-siblings by sires, based on the variance components of the sires, using a mixed model. When half-sibling groups are large enough and environmental factors are randomly distributed, inter-group differences can be attributed to genetic factors. These differences, represented as a variance, account for one-fourth of the additive genetic variance. By multiplying this value by 4, the total additive genetic variance is obtained:

$$h^{2}=\frac{4*\sigma_{IO}^{2}}{\sigma_{IO}^{2}+\sigma_{UO}^{2}}$$

*h*^2^—heritability coefficient

$\sigma_{IO}^{2}$—variance between sires; $\sigma_{UO}^{2}$—variance within sires;

Form for calculating the standard error of heritability in case of an unequal number of offspring by sires:

$${SGh}^{2}=4\sqrt{\frac{2\left(n.-1\right){\left(1-t\right)}^{2}{\left[1+\left(k-1\right)t\right]}^{2}}{k^{2}\left(n.-s\right)\left(s-1\right)}}$$

*SGh*^2^—standard error of heritability’s—number of sires *n*—total number of offspring *n_i_*—number of offspring by sires; *k*—correction coefficient for unequal number of offspring by sires; *n_i_* = *k* with an equal number of offspring by sires;

$$k=\frac{1}{s-1}\left(n.-\frac{\Sigma n_{i}^{2}}{n.}\right)$$

## 3. Results

### 3.1. Descriptive Analysis

Summary statistics related to evaluated parameters of development and slaughter characteristics, measured upon completion of the performance test, are shown in Table 1. The gilts reached a body weight of 100 kg between 200 and 291 days of age. The achieved result for age at the end of the test indicates satisfactory growth efficiency. The average values of backfat thickness were 9.830 mm at the first and 8.924 mm at the second measurement site. These values suggest a good level of fat cover and satisfactory carcass fat quality. The average depth of the *muscles longissimus dorsi* (MLD) at the end of the test was 53.40 mm, indicating well-developed muscle tissue. The observed range and variability in certain traits can be attributed to differences in rearing conditions, the genetic background of the animals, as well as the applied measurement methods and interpretation of results. The obtained results indicate a high level of efficiency in the tested animals and provide a solid basis for further selection aimed at improving production performance.

Table 2 shows LSM ± SE values of the tested traits through the effects of the following factors: farm, year of testing, and genotype of the animal. These data allow a detailed insight into the influence of systemic factors on the phenotypic manifestation of production traits in the tested gilts. The analysis of variances shows statistically significant differences between different farms, years, and genotypes, which indicate the importance of controlling these factors in order to achieve the most accurate estimates of production capabilities of the animal. In addition, the results suggest that the genotype and year of testing have a greater influence on the variability of some traits, while farm influence is more pronounced for other traits, such as backfat thickness and back muscle depth. These findings can contribute to an improvement in selection strategies, enabling more efficient adaptation of selection programs to specific production conditions.

Observing the farm as a source of variation, animals on farm 1 finish the test at an average of 222.03 days and are older than animals on farm 2 finishing the test at an average of 190.24 days, the difference between them being 32 days. Backfat thickness 1 is 2.378 mm larger on Farm 1 compared to Farm 2, while backfat thickness 2 is also higher on Farm 1 by 1.575 mm in comparison to Farm 2. Animals on farm 1 showed a lower back muscle depth compared to those on farm 2, with a difference of 4.895 mm. The youngest animals were recorded in the third year of testing, while the oldest were observed in the first year. The highest value for backfat thickness1 was registered in the first year, and the lowest in the third year. When considering the gilt genotype as a factor influencing variation, it was found that animals of the Duroc genotype were the oldest, while those of the Large White breed were the youngest. The lowest values of backfat thickness 1 and 2 were recorded in Landrace animals, and the highest in Duroc animals. Landrace animals showed the greatest back muscle depth, and Duroc animals the lowest. In addition to the above, it was observed that the genetic variability between different farms and breeds is significant, which indicates the possibility of improving breeding programs through the selection of specific genotypes. The results show that farm conditions, as well as the year of the test, can significantly affect the phenotypic traits, especially with regard to the test completion time and backfat thickness.

**Figure 1:**
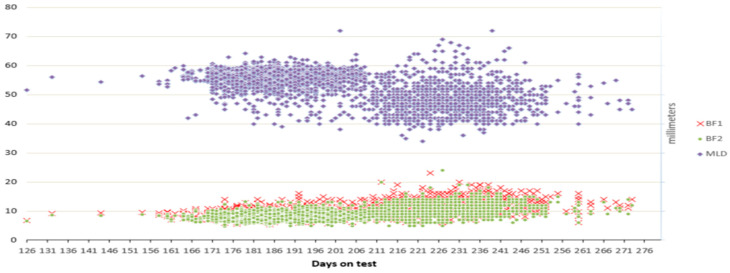
Variation of the observed traits in relation to test duration.

Figure 1 shows the variability of all analyses traits in relation to AET. A wide range of ages at the end of the test was observed, indicating the need to apply correction coefficients in this type of analysis. This correction can significantly contribute to a more accurate understanding of the factors that influence the development and performance of pigs, allowing a better adaptation of selection and management strategies.

Statistical significance of the fixed and regression effects considered in the models used for analyzing variability in growth and carcass quality traits in gilts, along with the coefficient of determination, is summarized in Table 3. The statistical significance of the fixed effects indicates that factors such as the farm, the year of testing, and the genotype of the animal have a significant influence on the phenotypic expression of the tested traits (*p* < 0.001).

The genotype of gilts had a highly significant effect (*p* < 0.001) on BF1 (backfat thickness 1), BF2 (backfat thickness 2), and MLD (depth of the back muscle), while it showed no significant influence on AET (age at the end of the test) (*p* > 0.05). The regression effects associated with the body weight of gilts at test completion also proved to be statistically significant, which confirms the importance of controlling these variables in the analysis of production performance parameters.

The year of testing had an effect on backfat thickness and muscle depth in gilts subjected to performance testing. Differences in final body weight were observed across the years, indicating variability in housing conditions and the potential influence of external factors. The results of the regression analysis show that an increase in body weight by 1 kg leads to an increase in backfat thickness of 0.086 mm at the first measurement site (BF1), 0.079 mm at the second site (BF2), and an increase in longissimus dorsi muscle depth (MLD) of 0.071 mm.

### 3.2. Genetic Parameters

Table 4 shows the components of variance for the observed traits during the performance test of gilts. These components provide insight into the relative contribution of genetic and environmental factors to the phenotypic variability of the studied traits. High values of variance may indicate a significant influence of heritability, while lower values often indicate greater sensitivity of traits to changes in environmental conditions. In addition, variance estimates represent a key step in the calculation of genetic parameters such as heritability that are essential for making selection decisions and improving selection programs.

The estimated heritability values for the four traits of interest (AET, BF1, BF2, MLD) are presented in Table 5. The highest heritability was recorded for backfat thickness at the first measurement site (BF1), with a coefficient of 0.3734 and a standard error of 0.0699, while a similar value was observed at the second measurement site (BF2) 0.3512 with a standard error of 0.0667. The heritability for age at the end of the test (AET) was 0.2647, with a relatively high standard error (0.1264). The lowest heritability was estimated for the depth of the longissimus dorsi muscle (MLD), amounting to 0.2320, but with a significantly lower standard error (0.0156), indicating greater precision in the estimate. The obtained values suggest that the analyzed traits in this study are characterized by a medium level of heritability.

## 4. Discussion

An observed phenotypic variability between different farms and breeds is significant as it indicates the possibility of improving breeding programs through selecting different gilt genotypes. These results highlight the importance of targeted selection strategies for enhancing the genetic potential in the analyzed populations of breeding gilts. In addition, the results show that conditions on the farm and years of research can significantly impact phenotypic traits, especially when the age at the end of the test and backfat thickness are in question. This suggests that a genetic improvement can additionally be optimized by improving the management of the environment through selection programs. Subsequent research may establish how specific conditions on the farm can impact genetic parameters and whether there is a possibility to standardize the methods of estimation of phenotypic and genotypic values.

The gilts reached a body weight of 100 kg at the age of 200 and 291 days (Table 1). The presented result for AET is better than the results presented in the research of many authors [5,16,17,18]; the aforementioned authors determined higher values for this trait. The results presented in Table 1 show values for average BF1 and BF2 of 9.830 and 8.924 mm, respectively. The reported average backfat thickness is consistent with the findings from previous studies [18,19]. The MLD depth at the end of the test averaged 53.40 mm, a result that is similar to some findings [19], but lower than others [18]. Moreover, the established differences between the studies can be partly explained by the different breeding conditions, the genotypes of the animals used, as well as the methods of measurement and interpretation of the results. However, the results achieved in this research indicate a high degree of efficiency of the tested animals and provide a basis for further selection in order to improve production performance due to the existing variability between measurements on farms.

A wide variety of ages at the end of the test was observed (Figure 1) emphasizing the need to apply correction coefficients in this type of analysis. Standardization of these values enables more precise estimation of the factors affecting the growth and performance of pigs by which use the accuracy of selection and strategy of management is being improved. The use of these corrections enables both researchers and farmers to compare more efficiently animals under different conditions securing more accurate estimation of genetic value. Moreover, improving the standardization methodology may contribute to a better understanding of the interactions between genetic and environmental factors in gilt development. Incorporating additional parameters such as microclimatic conditions, nutrition, and housing could enhance the understanding of phenotypic variability and facilitate the development of more efficient models for estimating breeding value.

The results indicate that the year of research and body mass significantly affect backfat thickness and back muscle depth in gilts. Regression analysis shows that an increase in body mass leads to an increase in backfat thickness and back muscle depth that confirms the relationship of these traits. These results emphasize the importance of precise management of selection and nutrition in order to optimize production characteristics in gilts.

The coefficients of determination (R^2^) show that the effects included in the models (farm, year, genotype, and body mass at the end of test) account for 44.3% and 73.8% variability of examined traits in gilt performance test (Table 3). The coefficient of determination (R^2^) represents a percentage of variability of traits that the model can explain. Higher R^2^ values suggest that models explain phenotypic variability correctly indicating that included factors significantly contribute to understanding the differences in growth and body quality. Differences in the coefficient of determination (R^2^) values between traits such as AET (age at the end of the test) and BF2 (backfat thickness) result from varying levels of influence of the analyzed factors. A higher R^2^ for AET indicates greater predictability due to management practices, while a lower R^2^ for BF2 suggests that much of its variability is not fully captured by the model, as it is affected by complex interactions between genetic and environmental factors. Increasing R^2^ for BF2 could be achieved by including additional covariates in the model. These results are crucial for improving the selection procedures and optimizing the production conditions on the farms. By integrating these findings into breeding strategies, a genetic improvement can be enhanced by which a more efficient and more productive system of production can be realized. Subsequent research could analyze in what way the external effects could be more precisely modeled in order to improve selection strategies while including the genomic data could additionally contribute to improving the accuracy of estimation of genetic parameters.

The variance components obtained for the traits (BF1, BF2, MLD, AET) during the gilt performance test provide insights into the contributions of genetic and environmental factors to the phenotypic variability of these traits. High values of variance show an important genetic contribution while lower values indicate a higher influence of environmental factors. These data are crucial for a precise estimation of heritability and decision-making on selection. Since heritability is the ratio of genetic to total phenotypic variance, accurate estimation of variance components is essential for its reliable calculation. A proper understanding of variability of examined traits can contribute to optimizing the selection programs and improving the breeding strategies.

A direct genetic effect explained 26.4747% overall variability of gilt age at the end of the test, which is displayed in Table 5. This value of heritability is higher compared to the results reported [11,12,20]; it is closer to the values shown in the study [17]. Heritability coefficients of 0.3734 for BF1 and 0.3512 for BF2 indicate that these traits are highly heritable in the analyzed population. However, the obtained heritability values for BF1 and BF2 are lower compared to those reported in the studies [11,12,21]. On the other hand, the heritability coefficient for the depth of MLD (0.2320) exceeds the values obtained in the research [11,12,17], but it is significantly lower in relation to the data obtained in the study [22]. These differences in the heritability estimations between the studies emphasize the importance of taking into account differences in the genetic structure of populations, methodological approaches, and conditions in which trials were conducted, along with the interpretation of genetic parameters. In addition, variations in heritability can indicate the need for better modeling and inclusion of additional factors that can contribute to enhanced precision in estimating the heritability coefficients.

High heritability values in these traits indicate significant potential for genetic improvement through selection. Implementation of properly structured breeding programs directed to these traits can accelerate the genetic progress of gilt populations. Furthermore, proper management of external factors such as nutrition and housing conditions can contribute to additional improvement of performances, maximizing a genetic potential and optimizing production results. This emphasizes the need for an integrated approach that combines genetic selection with optimal management in order to achieve sustainable improvements in pig breeding. Further research could include more detailed analysis of genetic correlations between examined traits what would enable an even more efficient selection approach and improve the precision of estimation of gilt breeding values taking into account the fact that the accuracy of the estimation of heritability coefficients has an effect on the accuracy of estimation of breeding values.

In addition, significant differences in heritability between different studies can be a consequence of differences in population genetic structure, methodologies of research or conditions in which the studies were conducted. Later studies that would include a wider specter of genetic and non-genetic factors can give additional insight into the variability of these traits and enable an even more accurate approach to selection. The research extended to genomic selection and advanced statistical models can additionally improve selection strategies providing continuous progress of genetic improvement in the future gilts, i.e., economically important traits.

In conclusion, to achieve faster and more reliable genetic progress, it is recommended to implement the BLUP-AM (Animal Model) method for the estimation of breeding values. Based on the results presented in this study, it was determined that the analyzed traits (AET, BF1, BF2, and MLD) have high heritability coefficients and can therefore be rapidly improved in a population under selection. All of this could be utilized to enhance economically important traits in gilts.

In addition, faster genetic progress should also be pursued through the genetic evaluation of multiple farms simultaneously using the BLUP-AM method, as well as through the application of molecular-genetic methods in gilt selection. Numerous SNP (Single Nucleotide Polymorphism) chips have been developed [23], and genes involved in the expression of economically important traits in gilts have been identified, which could enable precise identification and more effective selection for these traits.

## 5. Conclusions

This research provides a vital insight into the factors that can impact phenotypic variability and the genetic potential of gilts in a performance test. The results obtained show that all the analyzed factors in models have a statistically highly significant effect on backfat thickness variability (BF1 and BF2), back muscle depth (MLD), and age at the end of test (AET). A body mass at the end of the test has a significant effect on backfat thickness and back muscle depth. The estimations of heritability for these traits (BF1, BF2, MLD, and AET) are within a moderate scope ranging from 0.2320 to 0.3734, which suggests the possibility of a rapid improvement in the gilt population that is being selected. Including the backfat thickness and back muscle depth into selection programs can contribute to improving performances taking into account their expressed genetic variability and heritability. Moreover, optimization of the housing conditions and nutrition can decrease the effect of environmental factors on trait variability and can contribute to achieving more uniform production characteristics. An appropriate selection of gilts with the highest genetic potential is crucial for obtaining better production results in the pig meat industry. Selection of gilts with the highest genetic potential can positively impact the economic profitability of pig meat production. Furthermore, faster and more reliable genetic progress could be achieved by applying the BLUP-Animal Model method for breeding value estimation, as well as through the use of molecular genetic tools such as SNP chips, which enable more precise selection for economically important traits.

## Figures and Tables

**Table 1:** Average measures and dispersion of the examined characteristics.

Variable	N	$\bar{x}$	SD	Min	Max
AET (day)	3664	200.291	22.021	126.000	287.000
AETC (day)		180.824	19.219	122.222	273.810
BF1 (mm)		9.830	2.320	5.200	23.000
BF1C (mm)		8.353	1.763	4.476	17.377
BF2 (mm)		8.924	1.982	4.700	24.000
BF2C (mm)		7.684	1.540	3.803	19.419
MLD (mm)		53.367	5.147	34.000	72.000
MLDC (mm)		53.787	5.182	34.491	72.361

AET—age of the end of test; AETC—corrected age at the end of test; BF1—backfat thickness 1; BF1C—corrected backfat thickness 1; BF2—backfat thickness 2; BF2C—corrected backfat thickness 2; MLD—depth of the back muscle; MLDC—corrected depth of the back muscle.

**Table 2:** Least squares means (±*SE*) of analyzed traits by farm, year, and genotype.

Variation Factor		AET (Day)	BF1 (mm)	BF2 (mm)	MLD (mm)
Farm	1	222.035 ± 0.473	11.843 ± 0.067	10.375 ± 0.065	49.354 ± 0.156
	2	190.245 ± 0.381	9.456 ± 0.054	8.800 ± 0.052	54.249 ± 0.126
Year	1	210.268 ± 0.675	11.026 ± 0.096	9.885 ± 0.093	49.902 ± 0.223
	2	203.074 ± 0.605	10.349 ± 0.086	9.396 ± 0.083	51.311 ± 0.200
	3	201.387 ± 0.702	10.083 ± 0.099	9.137 ± 0.096	53.987 ± 0.232
Genotype	1	205.050 ± 0.522	10.036 ± 0.074	9.038 ± 0.072	52.308 ± 0.173
	2	204.467 ± 0.573	9.943 ± 0.081	8.943 ± 0.079	52.309 ± 0.189
	3	205.212 ± 0.780	11.479 ± 0.111	10.438 ± 0.107	50.584 ± 0.258

AET—age of the end of test; BF1—backfat thickness 1; BF2—backfat thickness 2; MLD—depth of the back muscle.

**Table 3:** Statistical significance of fixed and regression effects on AET, BF1, BF2 and MLD.

Studied Traits	Farm	Year	Genotype	T		R^2^
				B	*p*	
AET (day)	***	***	ns	-	-	0.738
BF1 (mm)	***	***	***	0.086	***	0.567
BF2 (mm)	***	***	***	0.079	***	0.443
MLD (mm)	***	***	***	0.071	***	0.521

T—weight at the end of the test, B—regression coefficient, R^2^—determination coefficient AET—age at the end of the test; BF1—backfat thickness 1; BF2—backfat thickness 2; MLD—depth of the back muscle; ns = *p* > 0.05; *** = *p* < 0.001; coefficients of determination-R^2^.

**Table 4:** Variance components for observed traits in the performance test of gilts.

Variance Components	Trait			
	AET (Day)	BF1 (mm)	BF2 (mm)	MLD (mm)
Additive (σ^2^_A_)	42.92316	0.65224	0.58396	3.12976
Phenotypic (σ^2^_P_)	162.11429	1.74661	1.6627	13.48746

AET—age at test completion, BF1—backfat thickness at the first measurement site, BF2—backfat thickness at the second measurement site, MLD—depth of the longissimus muscle.

**Table 5:** Heritability values (*h*^2^) and heritability standard errors (*sh*^2^) for observed traits in the performance test of gilts.

Observed Traits	N	*h* ^2^	*sh* ^2^
AET (day)	3664	0.2647	0.1264
BF1 (mm)		0.3734	0.0699
BF2 (mm)		0.3512	0.0667
MLD (mm)		0.2320	0.0156

*h*^2^—heritability coefficient, AET—age at test completion, BF1—backfat thickness at the first measurement site, BF2—backfat thickness at the second measurement site, MLD—depth of the longissimus muscle.

## Data Availability

Data used in this research are available from the corresponding authors on request.
